# Supplementary material for: Evaluation of antimicrobial and antiproliferative activities of Actinobacteria isolated from the saline lagoons of northwestern Peru
Source: PLoS One. 2021 Sep 8;16(9):e0240946. doi: 10.1371/journal.pone.0240946 (PMC8425546; doi:10.1371/journal.pone.0240946)
Supplement: S1 Fig — (DOCX) [file pone.0240946.s001.docx]

**S1 Fig.**

**S1 Fig. Flowchart depicting the methodological strategy adopted in this study**
