# Supplementary material for: Evaluation of antimicrobial and antiproliferative activities of Actinobacteria isolated from the saline lagoons of northwestern Peru
Source: PLoS One. 2021 Sep 8;16(9):e0240946. doi: 10.1371/journal.pone.0240946 (PMC8425546; doi:10.1371/journal.pone.0240946)
Supplement: S2 Fig — Total ion chromatogram (TIC) of UHPLC-MS analyses for (A) Streptomyces sp. MW562807 extract and (B) control. (DOCX) [file pone.0240946.s002.docx]

**S2 Fig.**


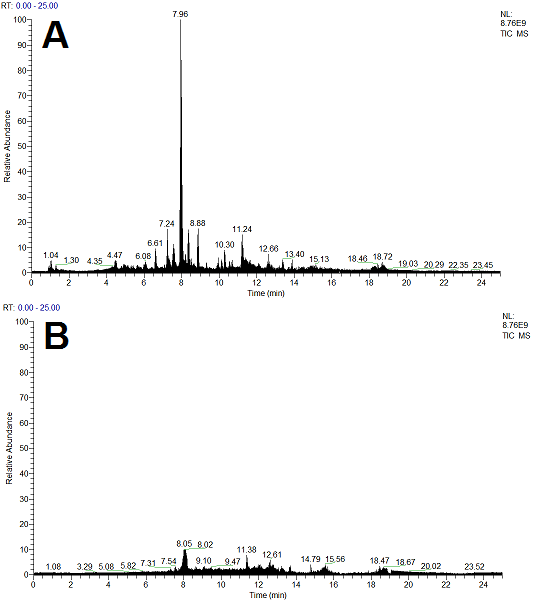


**S2 Fig.** Total ion chromatogram (TIC) of UHPLC-MS analyses for (A) *Streptomyces* sp. MW562807 extract and (B) control.
