# Supplementary material for: Evaluation of antimicrobial and antiproliferative activities of Actinobacteria isolated from the saline lagoons of northwestern Peru
Source: PLoS One. 2021 Sep 8;16(9):e0240946. doi: 10.1371/journal.pone.0240946 (PMC8425546; doi:10.1371/journal.pone.0240946)
Supplement: S3 Fig — MS/MS match between GNPS database (green) and Cholic Acid (1) from Streptomyces sp. MW562807 extract (black). (DOCX) [file pone.0240946.s003.docx]

**S3 Fig.**


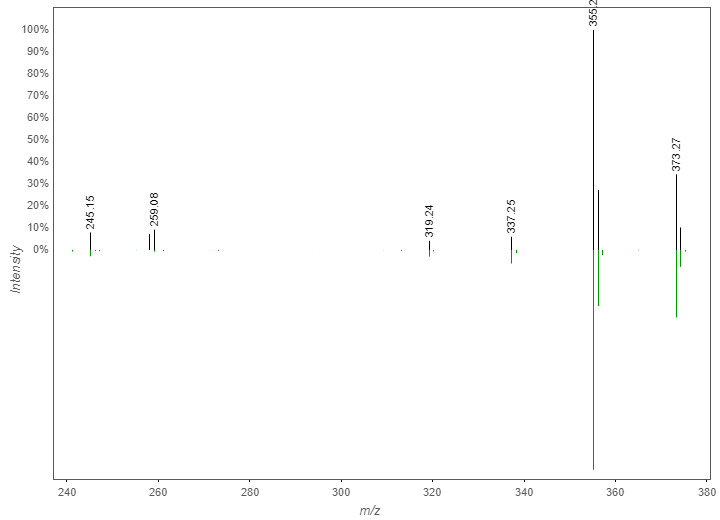


**S3 Fig.** MS/MS match between GNPS database (green) and cholic acid (**1**) from *Streptomyces* sp. MW562807 extract (black).
