# Supplementary material for: Evaluation of antimicrobial and antiproliferative activities of Actinobacteria isolated from the saline lagoons of northwestern Peru
Source: PLoS One. 2021 Sep 8;16(9):e0240946. doi: 10.1371/journal.pone.0240946 (PMC8425546; doi:10.1371/journal.pone.0240946)
Supplement: S4 Fig — Extracted ion chromatograms of m/z 426.32 for (A) Streptomyces sp. MW562807 extract and (B) control. (C) Mass spectrum of ion [M+NH4]+ m/z 426.3212 obtained for compound Cholic Acid (1) (error = -1.6 ppm) at 8.1 min. (DOCX) [file pone.0240946.s004.docx]

**S4 Fig.**


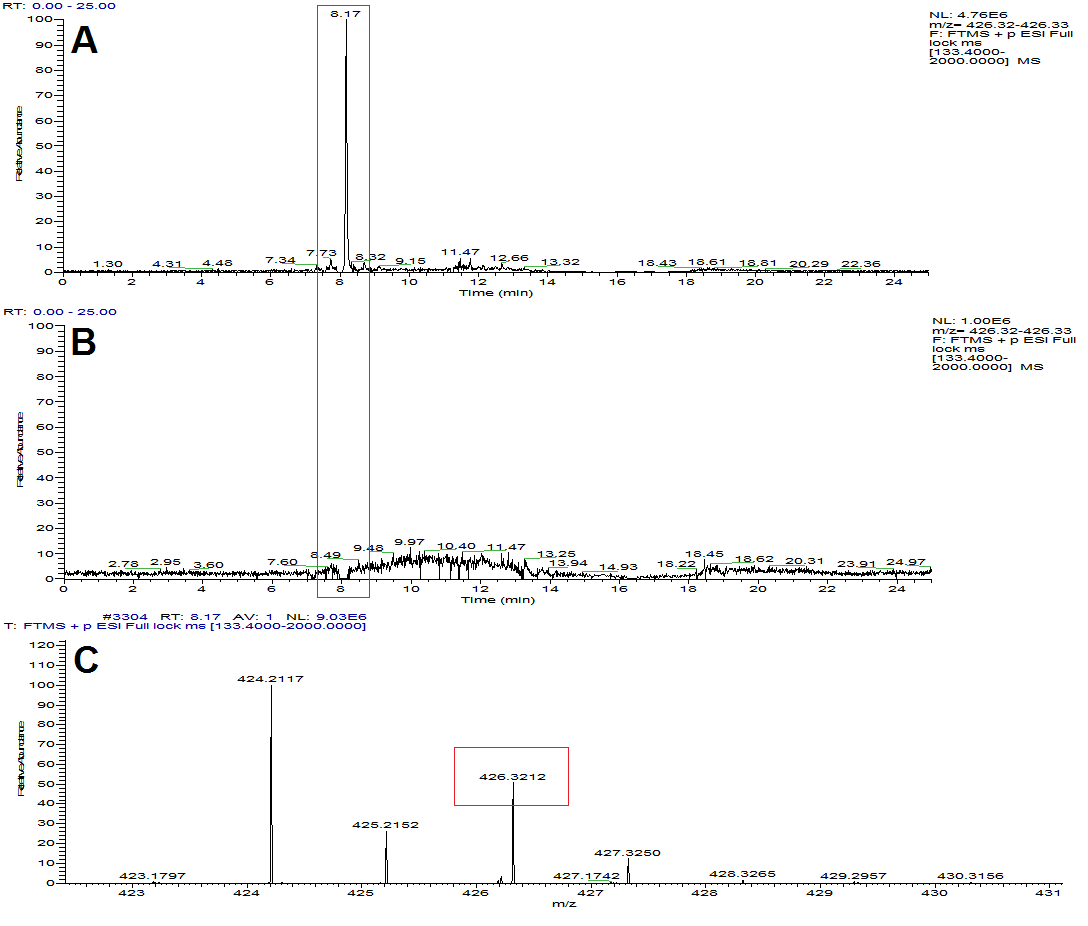


**S4 Fig.** Extracted ion chromatograms of *m/z* 426.32 for (A) *Streptomyces* sp. MW562807 extract and (B) control. (C) Mass spectrum of ion [M+NH_4_]^+^ *m/z* 426.3212 obtained for compound cholic acid (**1**) (error = -1.6 ppm) at 8.1 min.
