# Supplementary material for: Evaluation of antimicrobial and antiproliferative activities of Actinobacteria isolated from the saline lagoons of northwestern Peru
Source: PLoS One. 2021 Sep 8;16(9):e0240946. doi: 10.1371/journal.pone.0240946 (PMC8425546; doi:10.1371/journal.pone.0240946)
Supplement: S6 Fig — Extracted ion chromatograms of m/z 1157.63 for (A) Streptomyces sp. MW562807 extract and (B) control. (C) Mass spectrum of ion [M+H]+ m/z 1157.6373 obtained for Lobophorin A (2) (error = 0.1 ppm) at 7.9 min. (DOCX) [file pone.0240946.s006.docx]

**S6 Fig.**


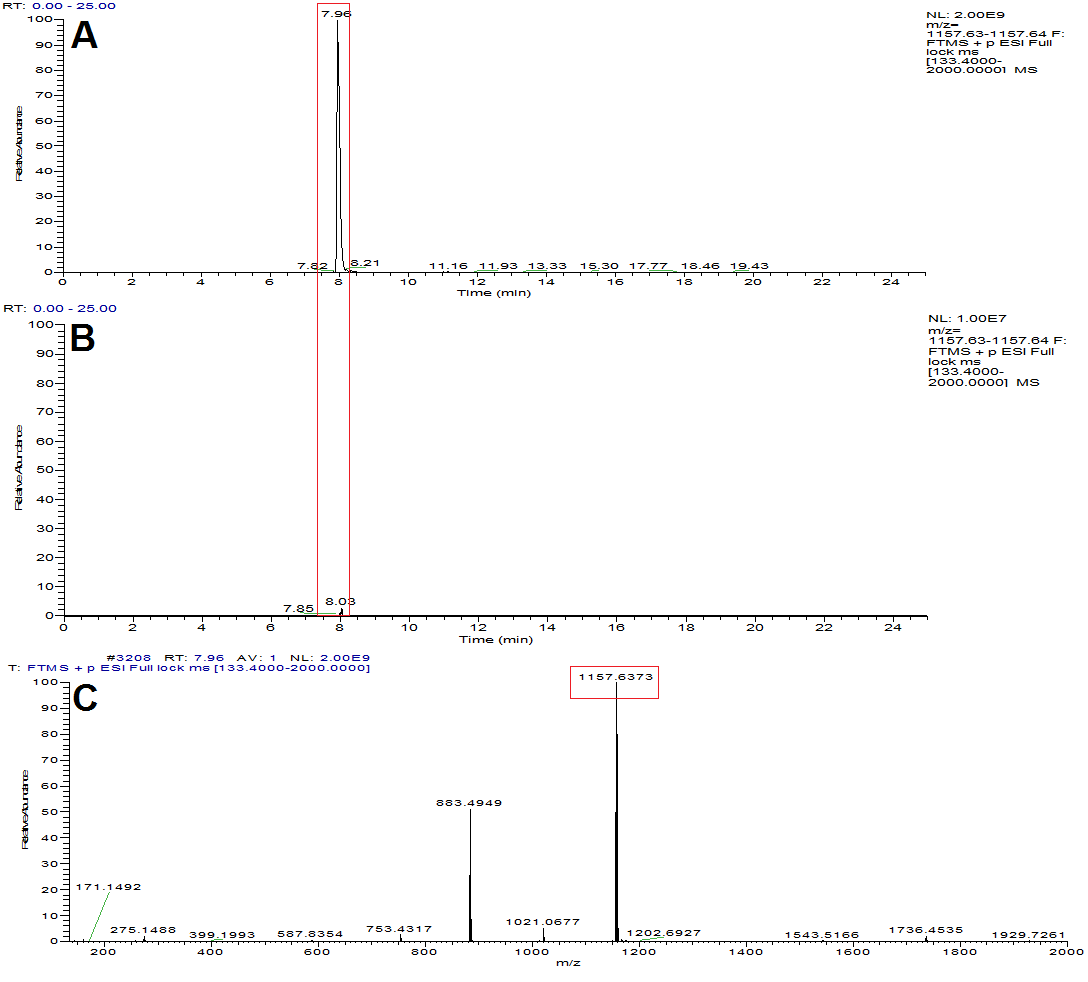


**S6 Fig.** Extracted ion chromatograms of *m/z* 1157.63 for (A) *Streptomyces* sp. MW562807 extract and (B) control. (C) Mass spectrum of ion [M+H]^+^ *m/z* 1157.6373 obtained for Lobophorin A (**2**) (error = 0.1 ppm) at 7.9 min.
