# Supplementary material for: Evaluation of antimicrobial and antiproliferative activities of Actinobacteria isolated from the saline lagoons of northwestern Peru
Source: PLoS One. 2021 Sep 8;16(9):e0240946. doi: 10.1371/journal.pone.0240946 (PMC8425546; doi:10.1371/journal.pone.0240946)
Supplement: S9 Fig — Extracted ion chromatograms of m/z 1173.63 for (A) Streptomyces sp. MW562807 extract and (B) control. (C) Mass spectrum of ion [M+H]+ m/z 1173.6321 obtained for Lobophorin K (5) (error = -0.4 ppm) at 7.2 min. (D) MS/MS spectrum of Lobophorin K. (DOCX) [file pone.0240946.s009.docx]

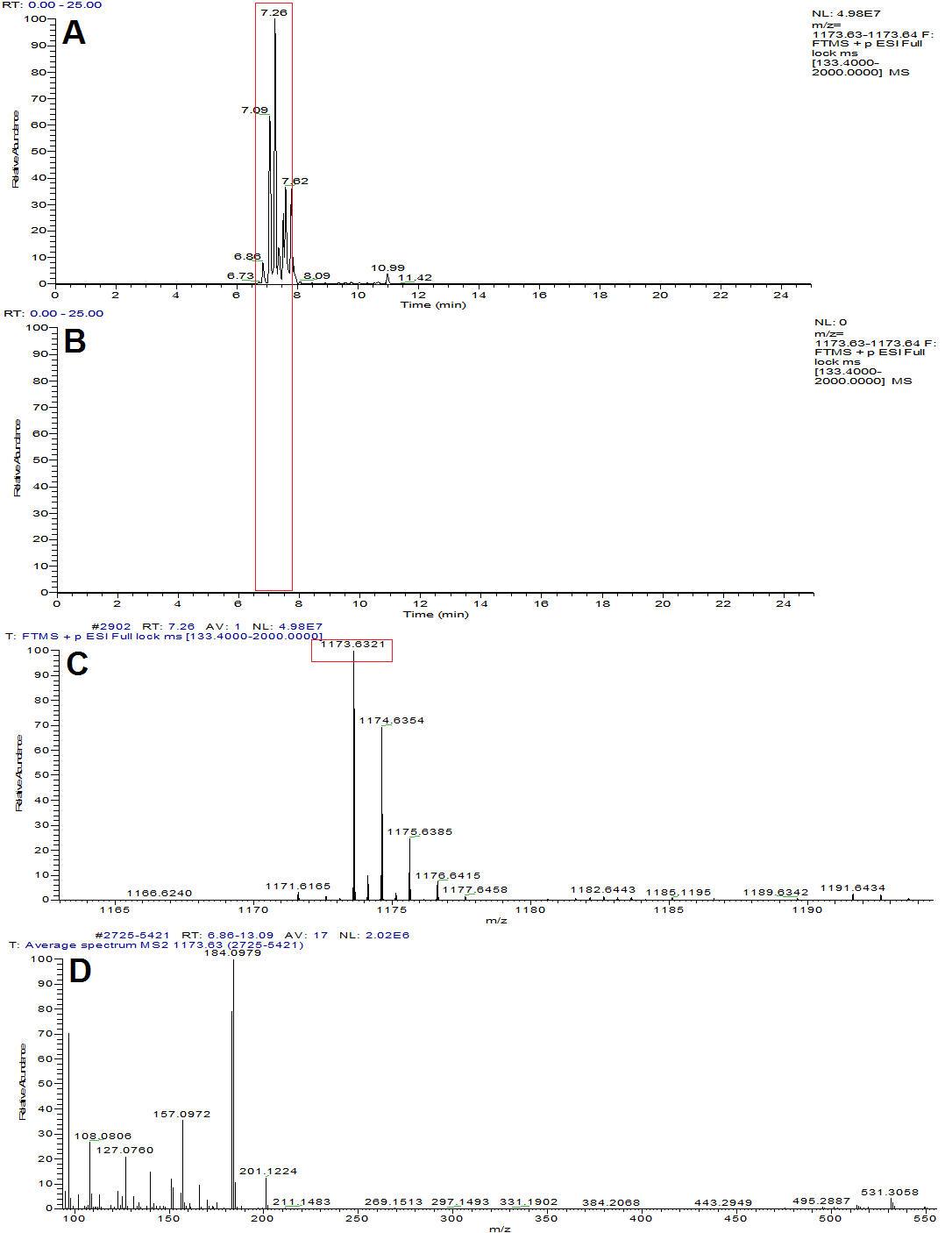
**S9 Fig.**

**S9 Fig.** Extracted ion chromatograms of *m/z* 1173.63 for (A) *Streptomyces* sp. MW562807 extract and (B) control. (C) Mass spectrum of ion [M+H]^+^ *m/z* 1173.6321 obtained for Lobophorin K (**5**) (error = -0.4 ppm) at 7.2 min. (D) MS/MS spectrum of Lobophorin K.
