# Supplementary material for: Evaluation of antimicrobial and antiproliferative activities of Actinobacteria isolated from the saline lagoons of northwestern Peru
Source: PLoS One. 2021 Sep 8;16(9):e0240946. doi: 10.1371/journal.pone.0240946 (PMC8425546; doi:10.1371/journal.pone.0240946)
Supplement: S10 Fig — Extracted ion chromatograms of m/z 197.11 for (A) Streptomyces sp. MW562807 extract and (B) control. (C) Mass spectrum of ion [M+H]+ m/z 197.1173 obtained for compound 6 (error = -2.0 ppm) at 7.2 min. (D) MS/MS spectrum of Compound 6. (DOCX) [file pone.0240946.s010.docx]

**S10 Fig.**


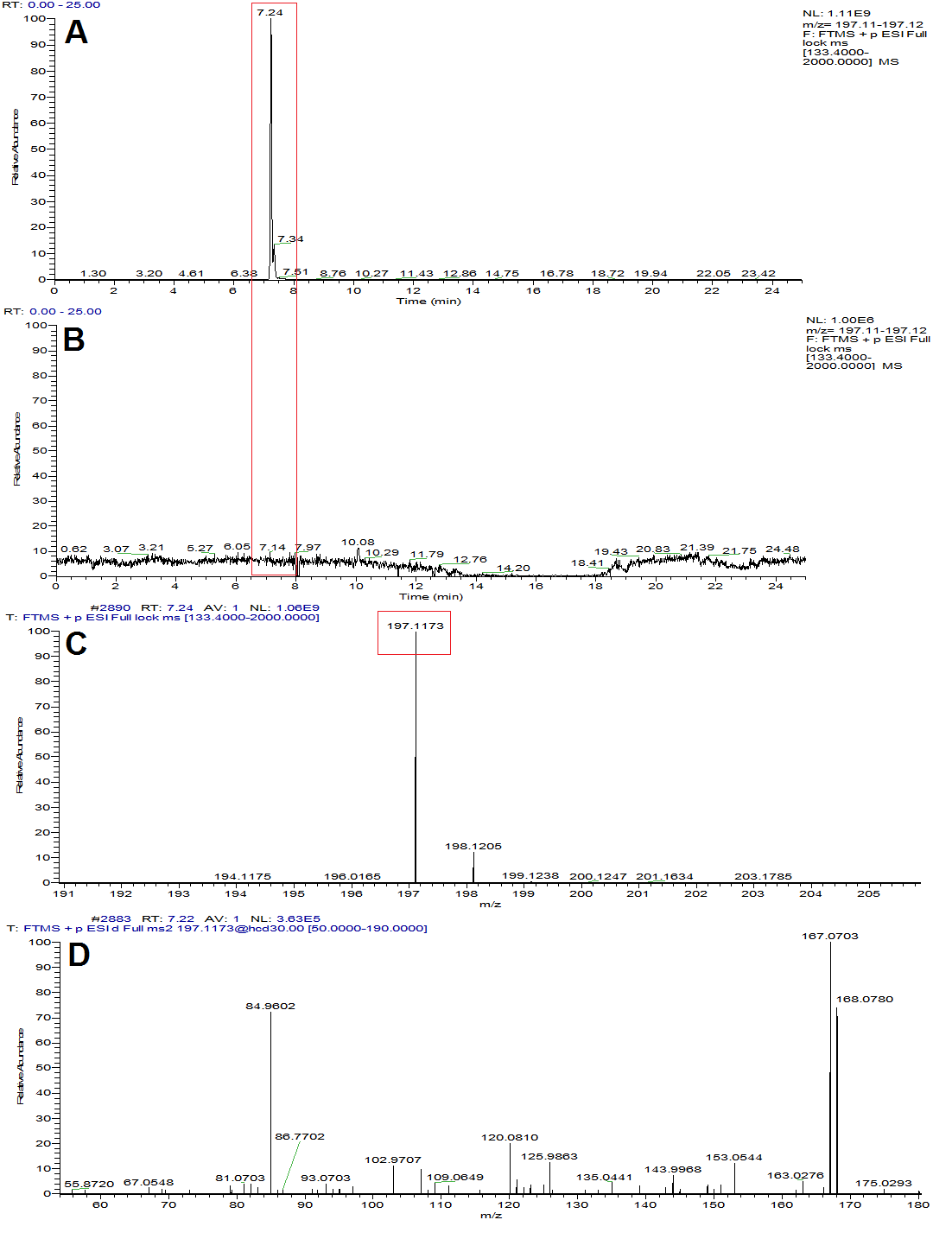


**S10 Fig.** Extracted ion chromatograms of *m/z* 197.11 for (A) *Streptomyces* sp. MW562807 extract and (B) control. (C) Mass spectrum of ion [M+H]^+^ *m/z* 197.1173 obtained for Compound **6** (error = -2.0 ppm) at 7.2 min. (D) MS/MS spectrum of compound **6**.
