# Supplementary material for: Evaluation of antimicrobial and antiproliferative activities of Actinobacteria isolated from the saline lagoons of northwestern Peru
Source: PLoS One. 2021 Sep 8;16(9):e0240946. doi: 10.1371/journal.pone.0240946 (PMC8425546; doi:10.1371/journal.pone.0240946)
Supplement: Attachment — Submitted filename: Response to Reviewers.pdf [file pone.0240946.s011.pdf]

Response to editor and reviewer(s)

Dear Academic Editor

Ph. D. Luis Angel Maldonado Manjarrez,

Emily Chenette

Deputy Editor-in-Chief

International Journal PLOS ONE

Dear Editor:

I wish to submit a research article for publication in *PLoS ONE*, titled **"Therapeutic potential Actinobacteria from northwestern Peru"**. The paper was coauthored by Nataly Ruiz Quiñones, Álvaro Tasca Hernandez, Ana Lúcia Tasca Gois Ruiz, Lucia Elaine de Oliveira Braga, Zhandra Lizeth Arce Gil, Luis Miguel Serquen Lopez, Jonas Henrique Costa, Taícia Pacheco Fill, Marcos José Salvador, and Fabiana Fantinatti Garboggini.

This study reports the isolation and phylogenetic analysis of several bacterial isolates from the saline lagoons from northern Peru, and their potential as producers of secondary metabolites with antimicrobial and antiproliferative activities. We found that the isolates of Actinobacteria *Streptomyces* spp. MW562814 , MW562805, and MW562807 may be new species; their importance lies in their antibacterial and antiproliferative potential. *Streptomyces* sp. MW562807 was the most promising for its activities. We believe that our study makes a significant contribution to the literature because our study revealed six biomolecules that can be further purified and characterized to develop them for various pharmaceutical applications.

Further, we believe that this paper will be of interest to the readership of your journal because it provides detailed insights into the field of natural sciences in terms of exploring new microhabitats that have remained untouched, in addition to carrying out a systematic and detailed study of unique extremophiles found in these environments, to extract compounds that are likely to have therapeutic potential against certain infectious diseases as well as some types of cancer.

This study was funded by the research agency of Peru (Concytec-Fondecyt) within the framework of the 041-01 call with the 190-2018 contract, it was the coauthors are grateful to the cooperation of the postgraduate programs in Genetics and Molecular Biology and Biosciences of the Biology Institute of the State University of Campinas. Also, Coordination for the Improvement of Higher Education Personnel - Brazil (CAPES), as the National Council for Scientific and Technological Development of Brazil (CNPq).

This manuscript has not been published or presented elsewhere in part or in entirety and is not under consideration by another journal. We have read and understood your journal's policies, and we believe that neither the manuscript nor the study violates any of these. There are no conflicts of interest to declare.

We would like to thank you and the reviewers for their comments and suggestions to improve the quality of our manuscript. Please find attached the revised version of our manuscript (PONE-D-20-29463) with the requested modifications. Specific responses to the reviewer's comments are given below in bold.
We hope that the manuscript is now suitable for publication in The International Journal PLOS ONE and if you need any additional information, please do not hesitate to contact us.

Sincerely

Rene Flores Clavo

Division of Microbial Resources

CPQBA-UNICAMP, Campinas-SP, Brazil

Postal code: 13081-970

Fone: +551921392896

Cellphone: +5519991640041

<u>Response to the Editor comments</u>

Comments from editor:

1. Please ensure that your manuscript meets PLOS ONE's style requirements, including those for file naming.

Reply: We have corrected the tittle, headings, authors' names, and affiliations according to PLOS ONE's style requirements.

2. We note that figure 1 in your submission contains map images which may be copyrighted. All PLOS content is published under the Creative Commons Attribution License (CC BY 4.0), which means that the manuscript, images, and Supporting Information files will be freely available online, and any third party is permitted to access, download, copy, distribute, and use these materials in any way, even commercially, with proper attribution. For these reasons, we cannot publish previously copyrighted maps or satellite images created using proprietary data, such as Google software (Google Maps, Street View, and Earth). For more information, see our copyright guidelines.

Reply: We have modified figure 1 in our manuscript. Now, the map has been removed and only the sampling sites photos remain.

3. Please ensure that reagents are described in sufficient detail for another researcher to reproduce the experiments described (source, product number, lot number).

Reply: We have made elaborated detailed protocols which are now published in protocols.io to make the requested information available.

4. Please provide additional information about each of the cell lines used in this work, including any quality control testing procedures (authentication, characterization, and mycoplasma testing).

Reply: We have added the requested information about the cell lines in the description to methods.

5. To comply with PLOS ONE submission guidelines, in your Methods section, please provide additional information regarding your statistical analyses.

Reply: We have added detailed information in the 2.7 Statistical analysis in the Methods section.

6. We suggest you thoroughly copyedit your manuscript for language usage, spelling, and grammar.

Reply: We have purchased the services of the suggested Editage editors for the language corrections.

7. We note that you have provided funding information that is not currently declared in your Funding Statement. However, funding information should not appear in the Acknowledgments section or other areas of your manuscript. We will only publish funding information present in the Funding Statement section of the online submission form.
Please remove any funding-related text from the manuscript and let us know how you would like to update your Funding Statement. Currently, your Funding Statement reads as follows:
"CONFLICT OF INTEREST STATEMENT
The authors declare no competing financial interests and the funders had no role in study design, data collection and analysis, decision to publish, or preparation of the manuscript."
Please include your amended statements within your cover letter; we will change the online submission form on your behalf.
Reply: We have removed the funding information from the acknowledgments section and put it on the Funding Statement in the Cover Letter.

8. Please include captions for your Supporting Information files at the end of your manuscript, and update any in-text citations to match accordingly.
Reply: We have added the captions information in the Supporting information section.

Comments from the nomenclature reviewers to be forwarded to the authors: The nomenclature reviewers agree with the proposed name and its etymology.
Reply: Thank you.

Response to the reviewer's comments

Reviewer #1: This manuscript describes the isolation of actinobacterial strains from a saline lagoon in Northwest Peru. The authors identified a selected group of actinobacteria by 16S rRNA gene sequencing and subjected the isolates to tests in order to determine whether they produce any bio-active compounds with activity against bacteria and various cancer cell lines. The extract from one of the most promising strains was evaluated via molecular networking, allowing for the prediction of compounds produced by this strain.
It is clear that the manuscript represents a large body of work, which also resulted in a rather lengthy manuscript. The authors would need to look at ways in which the manuscript can be shortened so that it is more streamlined. In addition, the authors also need to make sure that the way of citing references in the main text follow the journal's requirements.

Here are some additional comments/suggestions to the authors:

1. The manuscript will require language editing before it can be considered for publication.

REPLY: According to your suggestions, this manuscript has been sent to Editage Editors for language corrections.

2. Figure 1: the caption for image 'd' is not included in the figure legend.

REPLY: We have modified figure 1 in our manuscript. Now, the map has been removed and only the sampling sites photos remain. Now "d" is included in Figure 1.

3. Figure 2 can probably be presented as supplementary material.

REPLY: We have put Figure 2 to the Supplementary material section.

4. Page 8, section 2.2.: The authors described a very specific isolation protocol. This obviously would limit the amount and type of actinobacteria that would be isolated. What was the rationale behind using this method and were other isolation methods explored, but yielded no isolates of interest? In addition, many researchers often base their isolation protocol on the observed physicochemical characteristics of the environment the sample was collected from. Were any physicochemical analyses performed (e.g., sediment pH, level of salinity, presence of K, P, N, C, etc.)?

REPLY: We have developed and optimized a selective isolation protocol because the purpose of the research was to recover only actinobacteria, at the expense of Bacillus and Micrococcus, which has a faster growth. We have considered pH, solute concentrations, and other conditions from previous physicochemical analysis.

In addition, in this section and throughout the manuscript, when indicating a % solution, please indicate whether it is v/v or w/v, e.g., isolates were preserved in 20% (v/v) glycerol...

REPLY: Thank you. We have corrected those specifications about percentage.

5. Page 9, section 2.3.1: What was the final pH of the R2A-ASW broth used?

REPLY: We have corrected that specification o pH, which is 7.

6. Page 9-10, section 2.32: It is mentioned that modifications were made to the DNA extraction method reported by Pospiech and Neuman (1995), but no details are provided. Please expand on the modifications effected.

Line 161: The PCR protocol also calls for a large amount of DNA to be added. Is there any reason for that?

Reply: We have made elaborated detailed protocols which are now published in protocols.io to make the requested information available. Link: https://dx.doi.org/10.17504/protocols.io.bpvjmn4n

Line 166: What % agarose gel was used for the analysis of the amplicons?
Reply: We have made elaborated detailed protocols which are now published in protocols.io to make the requested information available. Link: https://dx.doi.org/10.17504/protocols.io.brrmm546

Lines 171-172: Which program was used to assemble to DNA sequences?
REPLY: The BioEdit 7.0 software was used for DNA sequences assembly and it is now written in the Methods section.

7. Page 11, line 186: What was the rationale behind the use of R2A broth for the production of bio-active compounds? Actinobacteria can produce numerous different compounds, a process that is greatly affected by the type of growth media used. Were different types of media tested, or did you solely focus on this medium?
REPLY: We have made elaborated detailed protocols which are now published in protocols.io to make the requested information available. R2A Broth, Nutrient Broth and SP2 Broth.

8. Page 11, lines 196-197: Here mention is made that the crude extracts were also analysed by LC-MS. Is this the same analyses reported later on in the methodology section (in which case the authors need to refer to the section) or was this a separate analysis (in which case more details are required)?
REPLY: Those analysis are the same. We have now corrected the reference in the Methods section.

9. Page 12, line 208: The wording of this sentence does not make sense – the 'three crude bacterial extracts' can't be 'tested as antimicrobial producers'. Best to say that they were tested for antimicrobial activity.
Reply: We have corrected that sentence and now it says "tested for antimicrobial activity.

10. Page 12, lines 211-212: What is meant by 'pre-coated microbial cultures'?
REPLY: We have corrected that sentence in the Methods section and now it just says that they were added.

11. Page 12, lines 217-218: Define the abbreviations 'MDRs' and 'UCI' – these have not been defined previously.
REPLY: We have detailed the definitions of those acronyms in the Methods section.

12. Throughout the manuscript, make sure that 'sp.' is not written in italics, e.g. page 13, line 244 – the 'sp.' should not be in italics.

REPLY: We have now corrected the italics in the "sp" throughout the manuscript.

13. Page 13, lines 244-245: It is indicated that the **MW562807** extract was diluted 1 in 10 in methanol, but there is no indication as to the final concentration of the extract. Please indicate, e.g. 1mg/ml, 0.1mg/ml, etc.

REPLY: We have now specified the final concentration of 6.4 mg/mL of the MW562807 extract in the Methods section.

14. Page 15, section 3.1: The use of phenotypic characteristics to group actinobacteria is notoriously unreliable. Genera within a family may have very similar morphological features and you stand a chance of 'missing out' potentially unique/'rare' actinobacteria, e.g. members of Kitasatospora and Streptomyces share phenotypic features, similarly members of Pseudonocardia and Nonomuraea. Why not identify all 166 strains?

REPLY: We did not identify all 166 strains because we were identifying bacteria because the main objective was to know the bacteria with antimicrobial and antiproliferative activities.

15. Page 17: The text provided in this section is basically a repeat of what is presented in Table 3. Only highlight the most interesting rather than repeating what is already presented.

REPLY: We have now kept only the most interesting instead of than repeating the same as Table 3.

16. Page 18, line 317 and 324: 'Streptomyces' should be written in italics.

REPLY: We have now corrected that word.

17. Page 18, lines 321-325: Here the authors highlight the types of actinobacteria isolated from the different environments. Care should be taken here especially since the authors only made use of a single isolation approach, which will result in isolation bias. They therefore can't compare what they detected during their isolations to that of others – they can simply report it and mention that these were isolated under the experimental conditions applied.

REPLY: We have now specified that we just report those bacteria according to the conditions of the samples they were obtained.

18. Figure 3: A phylogenetic tree containing only the three selected strains (and related streptomycetes) is presented in this figure. It seems a bit out of place since the importance of these three strains only becomes clear once the results for antimicrobial and anticancer tests have been reported. This makes it

seem out of order. I would recommend amending the tree to include all the Streptomycete strains identified in the study and once the bio-activity has been reported, reference can be made to this figure. This also means that Table 3 can be shifted to supplementary material.
REPLY: The phylogenetic tree is has been edited considering a representative strains for each of the 13 identified sub-groups.

19. Page 20, line 364: No evidence has been provided to support the classification of the isolates as 'extremophilic'.
REPLY: We have now specified that they were considered as extremophilic since they are able to grow in all media (Artificial Sea Water - ASW) supplemented with 5% of NaCl.

20. Page 22, line 395: Note the spelling of 'doxorubicin' – please correct.
REPLY: Thank you. We have now corrected that spelling.

21. Page 22, line 398: It is mentioned that the extracts from 'three different media' were selected. This is not reported in the methodology section – please amend.
REPLY: We have now specified that we just report those bacteria according to the conditions of the samples they were obtained.

22. Page 30, line 544: Note the spelling of 'cholic' – please correct.
REPLY: Thank you. We have now corrected that word.

23. Page 18, line 333: incomplete surname provided of the reference Cortés-Albayay.
REPLY: Thank you. We have now corrected that reference. Now we are using Mendeley to manage references.

24. Page 36, reference 40: Not cited in the main text.
REPLY: Thank you. We have now corrected that citation. Now we are using Mendeley to manage references.

25. Page 37, reference 42: Not cited in main text or incorrect spelling of lead author.
REPLY: Thank you. We have now corrected that citation. Now we are using Mendeley to manage references.

26. Page 37, reference 48: Not cited in main text or incorrect spelling
REPLY: Thank you. We have now corrected that citation. Now we are using Mendeley to manage references.

Reviewer #2: The article "EVALUATION OF ANTIMICROBIAL AND ANTIPROLIFERATIVE ACTIVITIES OF ACTINOBACTERIA ISOLATED FROM THE SALINE LAGOONS OF NORTHWEST PERU" describes the isolation and antimicrobial and anticancer capacity of strains isolated from saline environments. Although some of the results are of interest the quality of the manuscript is not enough, and a careful revision of data presentation and English should be taken into account before publication. It is not clear at any point, why the three strains of Streptomyces have been selected for further analysis, it is said that they have better inhibition results, but it has not been shown any experiment with the other strains. The authors should clarify that the sampling was in agreement with Nagoya protocol (check comment from line 110).
REPLY: Thank you. We have now specified that sampling was carried out according to The Nagoya protocol in the Methods section.

Lines 30-31: Reconsider this sentence, it has no many sense as it is.
REPLY: Thank you. We have now corrected those lines.

Line 35: add "and /or"
REPLY: Thank you. We have added “and/or”.

Line 36: add "these bacteria" before "were identified"
REPLY: Thank you. We have added “these bacteria”

Lines 36-38: I imagine these numbers refer to abundance of strains but it is not clear here.
REPLY: Thank you. We have now edited that line in the Methods section. We now just mention the Streptomyces genera without expressing quantities to avoid confusions.

Line 38: Actinobacteria should be in italics
REPLY: Thank you. We have now corrected that word.

Line 60: Here should be a final dot instead of semicolon.
REPLY: Thank you. We have now corrected the punctuation.

Line 69: Italics
REPLY: We have now corrected the italics.

Line 77: give a reference.
REPLY: We have now provided the corresponding reference.

Line 110: The Nagoya protocol was signed by Peru in 2014 so, at least the sampling of 2015 should be authorised by the goverment.

REPLY: We have initiated the corresponding procedures according to Peruvian laws we are currently obtaining the final documentation for the samplings from 2015 since the competent Governmental Entities and Normative Guidelines for the access to genetic resources specific to these sampling environments have been stablished recently in 2020, the print of the message is sent to the responsible entity with whom you are working to obtain the agreement for the use of the genetic resources.

De: Consultas OGACI <consultas@produce.gob.pe>
Enviado: martes, 1 de diciembre de 2020 17:43
Para: Elba Prieto Rios <dccbpa_temp10@produce.gob.pe>
Asunto: REGISTRO OACI-0016355-2020: Proyecto uso de recursos genéticos microbianos de fuentes de agua y sedimento de ambientes salinos

Estimada Srta. ELBA PRIETO – ESPECIALISTA DCCBPA:

Previo saludo, trasladamos el correo de la administrada Rene Flores a fin que pueda absolver la consulta según su competencia.

Agradecemos su pronta atención.

Atentamente.

Oficina de Atención y Orientación al Ciudadano
Oficina General de Atención al Ciudadano
Calle Uno Oeste 060 - Urbanización Córpac, San Isidro - Perú
Teléfono: (511) 616-2222 anexos 2461, 2462, 2436
www.produce.gob.pe
PERÚ Ministerio de la Producción

Line 112: it should be b, c and d, isn't it?

REPLY: Yes, thank you. We have now modified Figure 1.

Table 1: before was indicated that sampling was in July, clarified it. Indeed if it was in december the Nagoya protocol should be applied.

REPLY: Thank you. The procedures according to the Nagoya protocol were stablished in 2020 for the specific conditions and samples sites. We are currently obtaining the final documentation for the access to genetic resources.

Figure 2: correct "bacterial" and "extremophilic bacteria". But indeed this figure can be omitted.

REPLY: Thank you. We have now corrected in supplementary Fig 1.".

Line 126: it should be actinomycetes, not actinomyces, but indeed better if you use "Actinobacteria"

REPLY: We have now corrected that word.

Line 144: this amount means 2.43% not 5%, verified your data.

REPLY: We have now specified that ASW was supplemented with 5% of NaCl described protocol with reference 23.

Lines 276-279: These characteristics do not allow the classification into genus, you can group them into groups with similar characteristics, but not genus. This classification could be obtained after 16S rRNA gene analysis.
REPLY: We have modified that part in the Methods section and we classify them grouped in bacterial with similar characteristics.

Line 286: based on what? which strains were included in those groups? this should be further explained.
REPLY: We based this grouping on their genotypic similarities after the 16s rDNA gene alignment as shown in Table 2, and figure 2.

Line 288: What are the other 116 strains isolated?
REPLY: We isolated a total amount of 166 strains and then we could re-activate only 50 strains that were grouped into 13 clusters of actinobacteria identified by 16S rRNA.

Page 292: delete strain, and put "isolates" and "samples" in plural
REPLY: Thank you. We have now corrected that in the Results section.

Line 303: correct to "were similar" or "presented higher similarities with"
REPLY: Thank you. We have corrected that line.

Lines 300-317: it has no sense to give all the data here again if you give them in table 3, keep just the text or the table but not both. I recommend to keep the table.
REPLY: Thank you. We have now deleted in the table.

Line 317: Streptomyces should be in italics; delete "of the"
REPLY: Thank you. We have edited that word and that line.

Line 318: change "to" by "from"
REPLY: Thank you. We have edited that word.

Line 323: add a space before "has"
REPLY: Thank you. We have edited that word.

Lines 324 and 388: Streptomyces should be in italics
REPLY: Thank you. We have edited that word.

Line 328: correct the reference

REPLY: Thank you. We have edited that reference.

Figure 3: I do not understand why the authors has selected only three of their strains to construct the phylogenetic tree, a good analysis of their strains should include all the ones for which the 16s rRNA gene has been obtained, independently of their ability to produce secondary metabolites. The 16S rRNA gene sequences should be deposited in public databases, and their accession numbers included in the tree.

REPLY: Thank you. We made the phylogenetic tree considering the three. Representative strains from each group from 13 sequenced strains. Also, we have now added information about de deposited sequences in NCBI database.

Line 345: genus names should be in italics.

REPLY: Thank you. We have edited that word.

Line 353-356: this is not true, that is only true for the strain M-92, but not for the other two strains represented in the tree.

REPLY: Thank you. We have now corrected that information in the Results section.

Line 379: check the reference

REPLY: Thank you. We have now corrected the reference.

Line 401: delete "the"

REPLY: Thank you. We have deleted the "the".

Lines 405-407: these values are higher than 50, Shouldn't they be considered as inactive according to line 394 reference? This should be further discussed by the authors.

REPLY: Thank you. We have corrected and specified that information in the Results section.

Line 474: give details about the molecules marked as 1, 2 ,3, 4, and 5 in the figure.

REPLY: Thank you. We have now made a description specifying those molecules in the figure.

Line 541: it should be a comma after the first "study". Indeed one "study" should be replaced.

REPLY: Thank you. We have edited that line.

Line 542: correct "actinobacteria"

REPLY: Thank you. We have edited that word.

Line 550: delete the final dot after "potential"

REPLY: Thank you. We have now corrected the punctuation.
