# Supplementary material for: Evaluation of antimicrobial and antiproliferative activities of Actinobacteria isolated from the saline lagoons of northwestern Peru
Source: PLoS One. 2021 Sep 8;16(9):e0240946. doi: 10.1371/journal.pone.0240946 (PMC8425546; doi:10.1371/journal.pone.0240946)
Supplement: Attachment — Submitted filename: Response to reviewers.docx [file pone.0240946.s012.docx]

**Response to editor and reviewer(s)**

**Response to the Editor comments**

**Comments from editor:**

**1. Journal Requirements:**

Please review your reference list to ensure that it is complete and correct. If you have cited papers that have been retracted, please include the rationale for doing so in the manuscript text, or remove these references and replace them with relevant current references. Any changes to the reference list should be mentioned in the rebuttal letter that accompanies your revised manuscript. If you need to cite a retracted article, indicate the article’s retracted status in the References list and also include a citation and full reference for the retraction notice.

**Reply: We have corrected the references according to PLOS ONE's requirements.**

**Response to the reviewer’s comments**

**Reviewer #1:**

1. Page 2-3, lines 54-55 before, now 47-48 lines: ‘Three *Streptomyces* sp. strains’ sound cumbersome – consider changing the wording to ‘Three streptomycetes, strains MW562814,…’

**REPLY: Thank you. According to your suggestions, this manuscript we have corrected.**

2. Page 3, lines 59-60 before, now 51-52 lines: To streamline the sentence, consider writing ‘… and the multi-drug resistant *Acinetobacter baumannii* AC-972.’

**REPLY: Thank you. According to your suggestions, this manuscript we have corrected.**

3. Page 3, line 61: ‘…in the R2A medium was determined using a…’

**REPLY: Thank you. According to your suggestions, the manuscript has been corrected, and the abstract has been reduced for clarity, and this sentence in the manuscript text has been deleted.**

4. Page 4, lines 79-81 before, now 67-68 lines: Consider merging the two sentences to provide more flow to the information provided, e.g., ‘…were reported from saline soils of the ephemeral salty lakes in Buryatiya [4], while *Streptomyces*, *Nocardiopsis*, and…’

**REPLY: Thank you. According to your suggestions, this manuscript we have corrected.**

5. Page 4, lines 81-83. The sentence on biological activities seems out of place – I would recommend removing it.

**REPLY: Thank you. According to your suggestions, this manuscript we have removing it.**

6. Page 4, line 89 before, now 74: ‘…to the production of enzymes, which can…’

**REPLY: Thank you. According to your suggestions, this manuscript we have corrected.**

7. Page 8, lines 147, 156, 159-160 before, now 137,147, 150: It is conventional to refer to **16S rRNA** (as per other sections in the manuscript).

**REPLY: Thank you. According to your suggestions, this manuscript we have corrected.**

8. Page 8, line 158 before, now 148,149: What is meant by ‘represented’? Did you mean that the sequences were submitted to the EZBioCloud 16S database ‘Identify’ service?

**REPLY: Thank you. According to your suggestions, this manuscript we have corrected for “were added”.**

9. Page 9, line 162 before, now 152: ‘Mega’ should be written as ‘MEGA’

**REPLY: Thank you. According to your suggestions, this manuscript we have corrected.**

10. Throughout the manuscript: For any % solutions, please indicate whether they are v/v or w/v solutions.

**REPLY: Thank you. According to your suggestions, this manuscript we have corrected.**

11. Page 10, line 187 before, now 177,178: What is meant by ‘adjusted to a total volume of 200μl’? And is the ‘plate’ referred to a 96-well plate?

**REPLY: Thank you. According to your suggestions, this manuscript we have corrected “total volume of 200μl” and add 96-well plate.**

12. Page 13, lines 251-252 before, now 239,240: ‘…were used to group isolates into 42 filamentous and 8 non-filamentous bacterial groups’. Can’t use the information to ‘classify’, rather ‘group’, and since all the isolates were bacteria, best to keep it generic at this stage and use an inclusive term such as ‘bacteria’.

**REPLY: Thank you. According to your suggestions, this manuscript we have corrected.**

13. Table 2: The Family names should also be written in italics.

**REPLY: Thank you. According to your suggestions, this manuscript we have corrected.**

14. Page 14, line 283: Here is it indicated that MEGA7 was used, whereas in the methodology section it is indicated that MEGA6 was used – please correct.

**REPLY: Thank you. According to your suggestions, this manuscript we have corrected.**

15. Page 15, line 298 before, now 269 line: Remove ‘to’

**REPLY: Thank you. According to your suggestions, this manuscript we have corrected.**

16. Page 15, line 303 before, now 274: Replace ‘Actinobacteria’ with ‘actinobacterium’

**REPLY: Thank you. According to your suggestions, this manuscript we have corrected.**

17. Page 16, line 310 before, now 280 line: Replace ‘like’ (colloquial term) with ‘such as’ (more scientific).

**REPLY: Thank you. According to your suggestions, this manuscript we have corrected.**

18. Page 16, line 312 before, now 281 line: Did you mean to say that a member of the genus Pseudonocardia was also recovered during this study? In which case, the wording needs to change to reflect this: ‘…and plants, was also recovered in this study.’

**REPLY: Thank you. According to your suggestions, this manuscript we have corrected.**

19. Page 16, line 316 before, now 285 line (and throughout the manuscript): If there is no reference made to specific strains of a genus, then it is best to write out spp. as species. For example, here, it is best to write ‘…*Streptomyces* species as the most…’

**REPLY: Thank you. According to your suggestions, this manuscript we have corrected.**

20. Table 3: Replace ‘Isolates of *Streptomyces* sp.’ with ‘*Streptomyces* isolates’

**REPLY: Thank you. According to your suggestions, this manuscript we have corrected.**

21. Figure 3: It is recommended that the figure should be removed. The addition of all streptomycete isolates to Figure 2 negated the need for this figure.

**REPLY: Thank you. According to your suggestions, this manuscript we have removing it.**

22. Page 22: Be consistent in how the names of bioactive compounds are presented, e.g., shellmicin is written in both lower case and upper case in this section (see lines 430 and 436 vs 434 and 436) before, now (384,391,395,396,etc)

**REPLY: Thank you. According to your suggestions, this manuscript we have corrected Shellmicin A, …**

23. Figure 4: How does the inset information (*Streptomyces* B-81 and Control) relate to the figure presented?

**REPLY: Thank you. According to your suggestions, it was corrected for better understanding, than is indicated in the description in the figure 3.**

24. Page 25, line 463 before, now 423,424 lines: What is meant by ‘rolling in the GNPS platform database’?

**REPLY: Thank you. According to your suggestions, this manuscript we have added term “comparing in the GNPS platform database”.**

25. Page 25: Please see comment above about writing the names of bioactive compounds in lower case/upper case. Choose one and be consistent in its use.

**REPLY: Thank you. According to your suggestions, this manuscript we have corrected.**

26. Page 26, line 484: ‘gram-negative’ before, now 443 line needs to be replaced with ‘Gram-negative’. ‘Gram’ refers to the surname of the scientist who designed the staining technique and should therefore always be written with a capital letter.

**REPLY: Thank you. According to your suggestions, this manuscript we have corrected.**

27. Page 28, line 519 before, now 492 line: Remove the additional full stop after ‘sp.’

**REPLY: Thank you. According to your suggestions, this manuscript we have corrected.**

28. Page 28, line 527 before, now 498 line: Remove ‘one’.

**REPLY: Thank you. According to your suggestions, this manuscript we have corrected.**

29. Even though it was attempted to highlight additional language editing required, it is recommended that the revised manuscript should be evaluated once again before resubmission.

**REPLY: Thank you, if there has been a resignation of the major revision of the language in this new version committed.**

**Reviewer #3:**

1. It does not seem to be fully details on the culture conditions, time, and morphology of the colonies as these were recovered from the isolation plates and subcultured for further analyses. I think this is important should other authors who read the MS may also be encouraged to perform or follow the protocols indicated on their MS. If this information is missing, then I do not see how other authors may follow or identify their cultures.

**REPLY:** Thank you for your comment, we appreciate your evaluation very much. This information was described in the protocol on open access data base which was mentioned in the methodology section 132-133 (as described in a previously reported protocol <https://www.protocols.io/view/actinobacteria-collection-enrichment-and-isolation-brztm76n>). We describe the culture conditions, growth time and type of colonies morphology by microscopic observation.

2. After sequencing the 16S of the isolates, the authors indicate a size of 1,206 nt but the 16S rDNA gene is nearly 1,500 nt. Is there any particular reason why the gene was only partially sequenced and not completed? I am not saying that the full gene should be sequenced to accept the MS but an explanation within the MS would be useful on why 300nt were missing (technical problems? primers used? sequencing reactions?).

**REPLY: Thank you. The primers used amplify about 1000 bp covering regions V1 to V8, whose variability for genera level distinction is commonly used in diversity studies. It was added in the description of the phylogenetic tree in the specification if sequenced with primer 10F to 1492R, which resulted in cured sequences between 1206-1500 bp.**

3. Figure 3 indicates: Type strains within the genus *Streptomyces* and *Pseudonocardia* but the figure is not related nor include any Pseudonocardia, only Streptomyces!

**REPLY: Thank you. This figure has been replaced to include the genera *Streptomyces* and *Pseudonocardia*.**

4. In Figure 2 Streptomyces strain MW562807 falls in a subclade together with many other isolates. However, in that same tree MW562807 is also related to Streptomyces olivaceus and S. pactum. Have the authors checked if those two streptomycetes also show the same or similar biological activities to their isolate? Also since MW562807 is the most interesting strain, why the authors did not include on their study all related isolates to that strain acording to Figure 2? It would definitively be interesting to know if all the isolates within that subclade in Figure 2 do also show similar activities. Again, this is not meant to hold the MS any longer but a few lines within the conclusions or future work could be mention.

**REPLY: Thank you. According to your suggestions, this manuscript, were added three new references of Lobophorins compounds isolated from Streptomyces olivaceus strains (on lines 461-475), and it is presented that the strains that are reported for their biological activity from different lobophorins are also close *S. olivaceus* and *S. pactum*. In this study, a representative clade isolate was chosen to assess biological activities, however, we do not rule out this potential in future studies.**

5. **Conclusion**. It is clear that the authors have a good set of interesting strains which have been analysed in the MS. There is the MW562805 and related isolates but, in my view, MS562814 also deserves more comments. That isolate is not related to any type strain and it seems to be a putative novel species as it is not related to any other streptomycete in their phylogenetic trees. If the authors are to continue with this kind of studies, which one they think deserve more attention? The MSW562805 subclade or the MS562814?

**REPLY: Thank you. According to your suggestions, this manuscript we could not only discriminate a single strain, because all three have bioactive activities that is why the three will be taken to future work as we have mentioned in the conclusion within the lines (508-512). Taxonomic studies to characterize the strain MS562814 have been conducted by the group to describe a possible new species of the genus *Streptomyces*.**
